# An Overview of Ovarian Cancer: The Role of Cancer Stem Cells in Chemoresistance and a Precision Medicine Approach Targeting the Wnt Pathway with the Antagonist sFRP4

**DOI:** 10.3390/cancers15041275

**Published:** 2023-02-17

**Authors:** Lavanya Varier, S. Mohana Sundaram, Naisarg Gamit, Sudha Warrier

**Affiliations:** 1 Cuor Stem Cellutions Pvt Ltd., Manipal Institute of Regenerative Medicine, Manipal Academy of Higher Education (MAHE), Bangalore 560 065, India; 2 Division of Cancer Stem Cells and Cardiovascular Regeneration, Manipal Institute of Regenerative Medicine, Manipal Academy of Higher Education (MAHE), Bangalore 560 065, India

**Keywords:** cancer stem cells, ovarian cancer, CSC markers, Wnt pathway, sFRP4

## Abstract

**Simple Summary:**

Ovarian cancer is one of the deadliest cancers in women and is unfortunately detected only in its later stages, by which time it will have metastasized to different organs. One of the major determinants of the rapid proliferation and spread of cancer is the presence of cancer stem cells (CSCs) within tumors. In this review, we discuss the emerging markers of ovarian cancer with respect to CSCs and circulating tumor cells. Cancer stem cell population is regulated by the Wnt signaling pathway, which is described in detail. This is relevant to comprehend the significance of Wnt in regulating the stem cell-like properties of cancer. Cancer stem cells are also responsible for enhancing chemoresistant properties and metastatic potential. Therefore, for a successful treatment regime, it is important to factor in drugs that are specific to CSCs. By targeting the promoters of CSCs, such as the Wnt signaling pathway, it is possible to suppress these cells specifically. Herein, we describe an inhibitor of Wnt, secreted frizzled related protein 4, which could be used to successfully destroy the cancer stem cells of ovarian cancer.

**Abstract:**

Ovarian cancer is one of the most prevalent gynecological cancers, having a relatively high fatality rate with a low five-year chance of survival when detected in late stages. The early detection, treatment and prevention of metastasis is pertinent and a pressing research priority as many patients are diagnosed only in stage three of ovarian cancer. Despite surgical interventions, targeted immunotherapy and adjuvant chemotherapy, relapses are significantly higher than other cancers, suggesting the dire need to identify the root cause of metastasis and relapse and present more precise therapeutic options. In this review, we first describe types of ovarian cancers, the existing markers and treatment modalities. As ovarian cancer is driven and sustained by an elusive and highly chemoresistant population of cancer stem cells (CSCs), their role and the associated signature markers are exhaustively discussed. Non-invasive diagnostic markers, which can be identified early in the disease using circulating tumor cells (CTCs), are also described. The mechanism of the self-renewal, chemoresistance and metastasis of ovarian CSCs is regulated by the Wnt signaling pathway. Thus, its role in ovarian cancer in promoting stemness and metastasis is delineated. Based on our findings, we propose a novel strategy of Wnt inhibition using a well-known Wnt antagonist, secreted frizzled related protein 4 (sFRP4), wherein short micropeptides derived from the whole protein can be used as powerful inhibitors. The latest approaches to early diagnosis and novel treatment strategies emphasized in this review will help design precision medicine approaches for an effective capture and destruction of highly aggressive ovarian cancer.

## 1. Introduction

Ovarian cancer is the most lethal gynecological malignancy, with a 5-year survival rate of only 17% in the advanced stages [1]. Its prognosis is closely related to the stage at which the cancer is diagnosed [2]. Most patients remain relatively asymptomatic until the disease has spread beyond the ovaries and metastasized into the peritoneal space, producing non–specific symptoms such as dull abdominal pain or distension [3]. The rather occult nature of ovarian cancer makes it challenging to diagnose or screen the disease early. Although platinum chemotherapy is the most widely used treatment option, relapses remain high in these patients [4]. 

Ovarian cancer, despite its high lethality and low survival rate, does not have precise identification markers. Cancer stem cells are the key factor determining the drug response of the tumors and are usually identified by specific bonafide markers. In this review, we discuss the types of ovarian cancer and the existing diagnostic markers, chemo resistance conferred by CSCs and a unique panel for the identification of ovarian CSCs and circulating tumor cells. The role of Wnt in ovarian cancers, promoting stemness and metastasis, is a crucial factor in designing drug targets. An emerging antagonist of Wnt, secreted frizzled related protein 4 (sFRP4), has been recently found to be a promising agent to suppress CSCs. Could the functional peptides of sFRP4, in targeting ovarian cancers and their aggressive CSC population, evolve into a promising peptide drug panel for treating highly malignant and drug-resistant ovarian cancer? In this review, an overview of the existing challenges and evolving promising approaches and trends for ovarian cancer treatment is addressed.

## 2. Types of Ovarian Cancers

Most tumors from the ovary develop from one of the three components: the surface epithelium, germ cells and sex cord stroma. Epithelial ovarian carcinomas (EOC) account for the maximum number of cases and these are histologically subdivided broadly into five categories [5,6,7,8,9] as given in Table 1.

The definitive etiology and risk factors involved in the progression of ovarian cancer are not well studied but a few theories have been postulated, the majority having genetic links. Nulliparous women and those with longer reproductive years, i.e., early menarche and/or late menopause, are known to be at a higher risk for ovarian malignancy. On the contrary, multiparity and the use of oral contraceptive pills (OCPs), especially in pre- menopausal women and tubal ligation, prove to be protective factors. This is backed up by the incessant ovulation theory, which was hypothesized through a case control study conducted by Casagrande et al., where patients with factors accounting for “protected time”, i.e., longer periods of anovulation, through pregnancies and the use of OCPs had a significant reduction in the risk for developing ovarian cancer [10]. 

Among the epithelial ovarian cancers, the endometrioid and the clear cell type are known to be derived from ovarian endometriosis. A widely accepted theory is that of retrograde menstruation, which enhances iron-induced oxidative stress and in turn DNA damage. Some studies have even shown that gene mutations associated with endometriosis-associated ovarian cancers are normally found in the uterine and ovarian endometriotic epithelium [11]. High-grade serous ovarian carcinoma is considered the most aggressive type, accounting for the maximum number of deaths from ovarian cancer [12]. Women with BRCA1/2 germline mutations who underwent prophylactic salpingo-oophorectomy were found to have atypia of the tubal epithelium, raising the suspicion of the fallopian tube being the origin of serous ovarian cancers [13]. The retrograde menstruation theory holds good for the serous type too, with the DNA damage occurring more in the fimbriae of the fallopian tube as a result of chronic exposure to pooled blood in the pouch of Douglas. This process, along with the overexpression and the subsequent mutation of p53 at the mucosal cells of the fimbriae contribute to carcinogenesis [14].Table 1 elicits the distinct mutational profiles of the various subtypes of EOCs, suggesting that ovarian cancer is a highly heterogeneous disease. A more recent classification of OC is based on its grade and propensity for aggressive change, broadly categorizing it into Type I and Type II. Type I, which is the low-grade form, consists of low-grade serous, mucinous, endometrioid and clear cell whereas type II is the high-grade serous OC [15].

## 3. Existing Markers of Ovarian Cancer

Among the various cancer-detecting strategies, tumor markers have been used for decades as a starting point in the roadmap for cancer diagnosis. They are specific proteins and biomarkers released in blood in response to carcinogenesis and other inflammatory states. First identified by Bast et al. in 1981, CA-125 is the oldest and most widely used biomarker for epithelial ovarian cancer [16,17]. Although it is considered the standard tumor marker, it has low sensitivity in patients presenting in the early stages of the disease. Moreover, it is not a specific marker for ovarian cancer and is also observed to be elevated in endometriosis, menstruation, pelvic inflammatory disease, endometrioma, cirrhosis and pregnancy in some cases [12]. Studies showed that CA-125 was not often elevated in mucinous carcinoma, suggesting that it was not the most reliable marker for all histological subtypes of ovarian cancer [18]. CA-125 is also used as a prognostic tool to assess treatment response and recurrence, but over the years the urgent need for a more specific marker/combination of markers was emphasized, as the rise of CA-125 levels alone in an asymptomatic patient was not sufficient for diagnosis.

HE4 (WFDC2) is a glycoprotein belonging to the family of whey acidic proteins (WAP) and contains two WAP domains. It was initially isolated in human epididymis by Kirchhoff et al., but its presence outside the male reproductive system was noted eventually in further studies. Although it was weakly expressed in lung and colorectal adenocarcinoma, it was found to have significant expression in epithelial ovarian tumors, especially in the endometrioid subtype [19].

Statistical analysis showed a sensitivity of 72.9% for the HE4 marker alone when a comparative study between other markers such as CA-125, CA72-4, osteopontin, soluble mesothelin-related peptide and human epidermal growth factor 2 was performed by Moore et al., while Yanaranop and his team reported a specificity of 86% [16,20,21]. Studies also showed the efficacy of a combination of HE4 and CA-125 in the diagnostic algorithm and found an increased sensitivity at 92.9% [16,20]. Immunohistochemistry studies on tissues and oligonucleotide microarrays showed that the HE4 gene was strongly expressed in ovarian and endometrial lesions but not as much in endometriotic lesions [16,19]. This proved to be a significant differentiating factor from CA-125, which was overexpressed in endometriomas as well, enhancing the specificity of HE4 in ruling out benign ovarian lesions such as advanced endometriosis and ovarian endometrioma.

Moore and his team in 2009 proposed the Risk of Malignancy Algorithm (ROMA), which took into account HE4, CA-125 and the age of a patient presenting with a pelvic mass [22] and classified them as high risk or low risk (with the cut off for premenopausal women being 11.4% and 29.9% for postmenopausal women). Although it seemed to be a promising diagnostic tool, a study showed that it was not a satisfactory indicator to diagnose epithelial ovarian cancer in pre- menopausal women [23].

Prostatin (PSN), a trypsin-like protease normally found in prostatic and seminal fluids, was also studied as a potential biomarker when Costa et al. observed a significant overexpression in prostatin mRNA in epithelial ovarian cancers [24]. The combination of PSN and CA-125 has gained popularity over the years for its increased sensitivity (92%) and specificity (94%) in detecting early stage disease but the clinical use of PSN is yet to be rigorously assessed [12].

Transerythrin (TTR) has been found to be useful for the detection of stages I and II of epithelial ovarian cancer. Alpha fetoprotein (AFP) and beta HCG are used to detect the stages and treatment response of ovarian germ cell tumors. Inhibin A and B were found to be overexpressed in mucinous epithelial cancer and granulosa cell tumors [12,25].

## 4. Current Treatment Methods

Treating ovarian cancer requires a personalized and multi-disciplinary approach, taking into account the stage at which the patient is diagnosed (surgical staging) and their co-morbidities. A prospective study demonstrated a statistically significant difference in the benefit of chemotherapy and overall prognosis in patients whose cancer was optimally staged [26]. This reiterates the value of precise OC staging for future treatment assessment. 

Debulking cytoreductive surgery, which consists of total abdominal hysterectomy (TAH) + bilateral salpingo-oophorectomy (BSO) followed by three to six cycles of chemotherapy, is the initial step in the management of OC, recommended by the Gynecologic Cancer Inter-Group (GCIG) [27]. Nodal metastases are also seen in up to 28% of serous OC patients in early stages; thus, removal of the pelvic- and para-aortic lymph nodes is recommended [28]. Platinum alkylating agents such as carboplatin or cisplatin, the former being preferred due to milder side effects, and taxanes such as paclitaxel or docetaxel are the mainstays of adjuvant chemotherapy. They are administered intravenously or intraperitoneally, but hyperthermic intraperitoneal chemotherapy (HIPEC) has been found to be more effective at targeting residual tumor in stage III disease [29,30].

Unfortunately, 75% of patients are diagnosed for the first time in advanced stages (FIGO stages III and IV) of OC or patients have returned with recurrent disease that has metastasized extensively. This has prompted physicians to adopt a more aggressive approach, i.e., pre-operative imaging followed by maximal cytoreduction. A meta-analysis by Bristow et al. [31] elucidated that, among all other prognostic variables, maximal cytoreduction and surgical follow up had the most significance in assessing the median survival of a patient with advanced OC. Along with primary debulking, total omentectomy is essential due to the high prevalence of occult metastasis in the supracolic omentum. Although platinum agents have shown considerable effect in the medical treatment of OC, at least half of patients require radical and supra-radical surgery. If the tumor is found to be unresectable in some patients intra-operatively, palliative chemotherapy alone is the treatment of choice [28,31]. Response and sensitivity to platinum chemotherapy is measured by disease remission in a platinum-free interval of 6-12 months. In platinum sensitive patients with recurrence and metastasis, combined chemotherapy with the same drugs is used. However, the limited chemotherapy options for platinum-resistance and refractory states, with the combination of trabectedin and liposomal doxorubicin being one such, led to the discovery of targeted immunotherapy and PARP inhibitors [32,33].

### 4.1. PARP (Poly(ADPribose) Polymerase) Inhibitors

The role of PARP inhibitors as a single therapeutic agent is most beneficial in HGSOC patients with BRCA mutations. PARP 1 plays an important role in DNA single stranded breakage (SSB) and double stranded breakage (DSB) repair through base excision repair mechanisms. BRCA genes, among other proteins in normal cells, are involved in the homologous recombination repair (HRR) of DSB. The inhibition of PARP in the presence of BRCA 1 and 2 mutations results in defective HRR and DSB repair leading to genomic instability and cell death. This is referred to as ‘synthetic lethality’, wherein the inactivation of two pathways simultaneously evokes a strong and selective apoptotic response in cancer cells [32,34]. The FDA-approved drugs at present are olaparib, niraparib and rucaparib [35]. Niraparib and its combination with some anti-angiogenic agents have proven to be effective in platinum-resistant states [36]. 

### 4.2. Anti-Angiogenic Therapy

The vascular endothelial growth factor (VEGF) pathway is the most common target for angiogenesis inhibition in many cancers including OC. Bevacizumab (avastin) is a monoclonal antibody that targets the VEGF pathway and is approved for use in advanced OC patients [37]. Over time, resistance to VEGF inhibitors was observed due to alternate angiogenic pathway adaptations. This is partially overcome when bevacizumab is used as an adjunct with PARP inhibitors and platinum chemotherapy [38].

### 4.3. Immunomodulators (Pembrolizumab, Dostarlimab)

Epithelial OC is considered an inflamed tumor, one that has a high percentage of CD8+ T cells and high PD 1 expression [39]. This plays a key role in suppressing host tumor immunity, which suggests that using immune checkpoint inhibitors that target PD1/PD L1 or cytotoxic T lymphocyte-associated protein 4 (CTLA-4) shows promising results in advanced OC with DNA mismatch repair deficiency (dMMR) [40].

### 4.4. Radiotherapy

**Figure 1:**
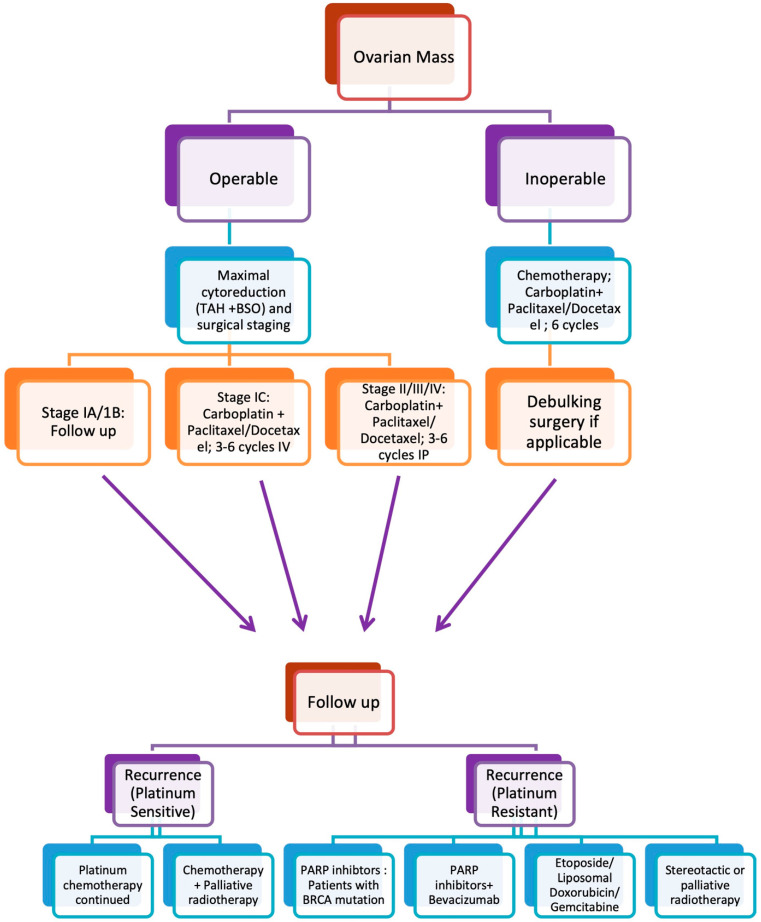
Summary of the widely used treatment strategies for ovarian cancer based on surgical staging and other treatment options in the case of disease recurrence.

Although ovarian cancer is known to be radiosensitive, the role of whole abdominal radiotherapy (WAR) is limited in standard care after the introduction of platinum-based chemotherapy [41]. This is attributed to post-radiation toxicity and the higher success rate of chemotherapy. Currently, stereotactic and palliative radiation are reserved for some patients with advanced, relapsed and non-resectable disease as salvage therapy. The primary benefit is symptom relief and marginally prolonged life expectancy [42]. The present treatment strategies for ovarian cancer are elaborated in Figure 1.

Complete remission is achieved with platinum chemotherapy and debulking surgery in a majority patients, but most of them experience a relapse [43]. Despite maintenance therapy with PARP inhibitors and targeted immunotherapy showing promising results in advanced disease [44], the long-term disease-free interval of ovarian cancer lasting around 10 years is still stagnant at 18% [45]. This shortcoming reflects the lack of a sufficient precision medicine approach addressing the molecular topography and chemo resistance in ovarian cancer cell lines. 

## 5. Chemoresistance and Cancer Stem Cells in Ovarian Cancer

Cancer stem cells (CSCs) are a small subset of the stem cell population with properties such as self-renewal, differentiation and resistance to apoptosis, similar to tissue stem cells [46]. The distinguishing property of CSCs is that they have an exponentially higher potency to seed a new tumor and cause continued tumor progression compared to non-CSC tumor bulk. They remain in a quiescent state and are unaffected by chromosomal aberrations and ageing [47]. Cancer stem cells are characterized by resistance to radiation by virtue of their advanced DNA repair mechanism, conferred by their elevated MDM2 activity, which is a transcriptional target of p53 [48].

The presence of tumor-initiating cells in malignancies was initially discovered by Lapidot et al. in 1994 when they found the increased expression of certain cell surface markers (CD34+, CD38+) in relation to acute myeloid leukemia in an immunocompromised state [49]. Over time, the significant presence of CSCs in solid tumors became evident in metastatic sites such as malignant ascites. Analysis on ovarian tissue using immunohistochemistry for pluripotency-associated cell surface markers revealed a small sub-population of Nanog-positive cells in ovarian surface epithelium (OSE) [50]. This lent support to the theory that these are cancer stem cells, dormant in ovarian surface and tubal epithelium. Incidentally, the majority of ovarian cancer cells originate from OSE, validating the prominent role of CSCs in the progression and recurrence of ovarian cancer [51]. 

Cancer stem cells are identified by their unique profile of cell surface and intracellular markers. The panel of ovarian CSC markers that are reported include CD24, CD44, CD117, CD133, ALDH1, ABC transporter proteins, EpCAM, Nestin, Oct4, Nanog, Sox2, SSEA4 and SCF. Of these, CD24, CD44 and CD133 are a few of the prominent markers. CD24+ cells in ovarian cancer exhibit anoikis resistance, higher tumor growth, colony formation, EMT phenotype and possess stem-like properties such as self-renewal. CD133 expression is associated with poor prognosis and is also implicated in increased platinum resistance and metastasis. Nanog expression also modulates platinum resistance and EMT in ovarian cancer. A comprehensive list of ovarian-specific CSC markers and their role is summarized in Table 2. 

The proliferative potential of CSCs is regulated by the tumor microenvironment and multiple pathways. Heterogeneity in the tumor microenvironment is a classic feature of ovarian cancer and the distinct mutational profile of epithelial ovarian cancer is what makes it unique and challenging among other cancers. The clonal evolution theory was proposed in 1976 [89], where longitudinal samples of a single patient’s OC cells before surgery and after recurrence, in ascitic cells from peritoneal dissemination, elicited a wide range of genomic diversity in tumor spheres at different locations [90]. The cellular plasticity that these tumors exhibit is primarily initiated in CSC niches and is the driving force for chemotherapeutic failure and disease progression in ovarian cancer. This occurs through phenotypic cellular changes brought about by interaction between the tumor cell and surrounding stroma or stochastic variations [27].

Multiple regulatory pathways such as Wnt, STAT3, Hedgehog, BMI1, Notch and NF-κB are abnormally activated and thus aid in the self-renewal of CSCs in ovarian cancer [91,92]. The dysregulated cell cycle and hyper-proliferation of CSCs are governed by modulators such as ß-catenin, MAPK, NF-kB, PAX6, FOXO3 and STAT2 [93,94]. For combating ovarian cancer, drugs are being designed to target self-renewal signaling pathways that can restrain the survival, differentiation and replication of stem cells. A few such drugs in clinical use are ipafricept (OMP-54F28) and DAPT (GSI-IX), which target the Wnt ligand (Wnt pathway) and γ-secretase (Notch pathway), respectively [95,96]. Besides ipafricept, there are several other potential therapeutic drugs that target the Wnt signaling pathway and are currently under clinical trials for multiple cancers. These include LGK-974 (WNT974; porcupine inhibitor) [97,98], ICG-001 (PRI-724; blocks the binding of β-catenin to CREB binding protein) [99], vantictumab (OMP-18R5; blocks Wnt/Fzd binding by targeting Fzd) [100].

## 6. Circulating Tumor Cells

The presence of circulating tumor cells (CTCs) in ovarian cancer is gaining clinical relevance [101]. Circulating tumor cells are cells that originate from the primary tumor bulk but break off into blood/lymphatic circulation. In recent years, studies established the hematogenous spread of ovarian cancer and this has attracted interest to develop non-invasive CTC-based tests to identify reliable diagnostic and prognostic markers for ovarian cancer [102]. Recently, one such study was conducted by isolating CTCs from 38 patients with advanced high-grade serous ovarian cancer. The results showed the higher expression of cancer stem cell markers CD24 and CD44 along with EMT-associated markers such as TIMP1 and CXCR4. The presence of these markers supports the hypothesis that CTCs exhibit molecular plasticity by expressing epithelial, mesenchymal and stem cell-like markers. This property helps in CTC migration and survival under hostile microenvironments and chemotherapeutic assault [103]. There is a correlation between CD24 expression in primary ovarian cancer tissue and lymph node metastasis, indicating the role of stem cell markers in metastasized OC [52]. Interestingly, tumor-derived exosomes isolated from patient plasma also showed a threefold increase in CD24 as identified by the Exosearch chip method [104]. These findings throw light on an exciting approach to develop efficient CD24-based non-invasive tests from CTCs in diagnosing and monitoring aggressive and metastatic ovarian cancer.

## 7. Role of Wnt Pathway and EMT in Ovarian Metastasis

Wnts are cysteine-rich glycoproteins secreted in the extracellular matrix. The Wnt pathway is a highly conserved evolutionary system, existing across species from invertebrates to mammals, and is responsible for various cellular processes such as proliferation, differentiation, apoptosis, tissue homeostasis and stem cell renewal [105]. Nineteen Wnt proteins couple with receptors and co-receptors in over seven protein families among which the frizzled protein functions as the principal Wnt receptor in mediating specific pathways [106]. Three fundamental Wnt pathways have been studied extensively: the canonical pathway, the non-canonical planar cell polarity pathway and the non-canonical Wnt/calcium pathway. 

**Figure 2:**
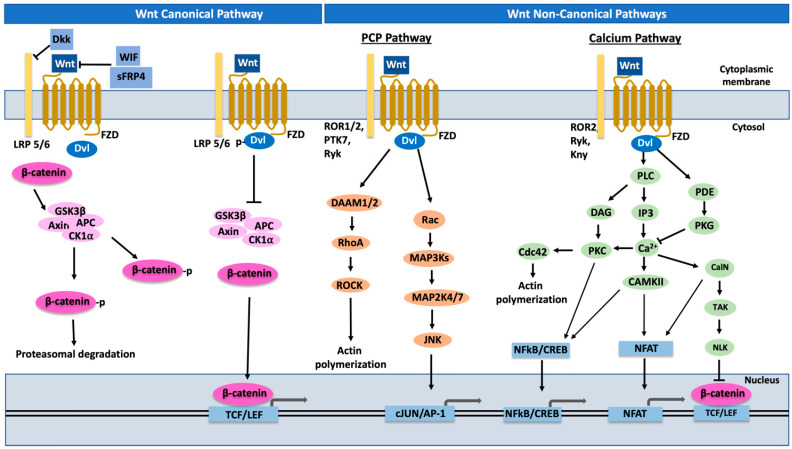
Wnt signaling pathways. Wnt canonical (β-catenin dependent) and Wnt non-canonical (Planar cell polarity and Calcium) pathways. AP-1, Activation protein 1; APC, Adenomatosis polyposis coli; Ca^2+^, Calcium; CalN, Calcineurin; CAMKII, Calcium-calmodulin-dependent kinase II; Cdc42, Cell division control protein 42 homolog; CK1α, Casein kinase 1 alpha; CREB, cAMP response element-binding protein; DAAM1/2, Disheveled-associated activator of morphogenesis 1/2; DAG, Diacylglycerol; Dkk, Dickkopf; Dvl, Disheveled; FZD, Frizzled; GSK3β, Glycogen synthase kinase 3β; IP3, Inositol 1,4,5-triphosphate; JNK, c-Jun N-terminal kinase; LEF, Lymphoid enhancer-binding factors; LRP 5/6, Low-density lipoprotein receptor-related protein 5 or 6; MAP2K 4/7, Mitogen-activated protein kinase kinase 4/7; MAP3Ks, Mitogen-activated protein kinase kinase kinase; NFAT, Nuclear factor of activated T cells; NFκB, Nuclear factor kappa light chain enhancer of activated B cells; NLK, Nemo-like kinase; PDE, Phosphodiesterase; PKC, Protein kinase C; PKG, Protein kinase G; PLC, Phospholipase C; Rac, Ras-related C3 botulinum toxin substrate; RhoA, Ras homolog family member A; ROCK, Rho Kinase; ROR2, RAR-related orphan receptor 2; Ryk, receptor-like tyrosine kinase; sFRP, Secreted frizzled related protein; TCF, Transcription factors T cell factor; WIF, Wnt inhibitory factor; Wnt, Wingless-type MMTV integration site.

The canonical Wnt pathway is dependent on the dual function protein, beta catenin. In the absence of a Wnt ligand, the cytoplasmic beta catenin is phosphorylated by the actions of adenomatous polyposis coli (APC), Casein kinase 1 alpha (CK1α) and Glycogen synthase kinase 3β (GSK3β) present in Axin (a scaffold protein that constitutes the destruction complex) [93,107]. This results in the continuous degradation of β-catenin via the ubiquitin-proteasome pathway. The activation of the Wnt ligand occurs when it binds to the surface co-receptors comprising seven transmembrane frizzled proteins and low-density lipoprotein receptor-related protein 5 or 6 (LRP 5/6). This interaction results in the activation of the protein Disheveled (Dvl), which ultimately leads to the inactivation of the destruction complex by GSK3β inhibition. The stabilization of β-catenin in signal transduction is a key component of the Wnt/β catenin pathway functioning and this is achieved by Wnt ligand activation wherein the stabilized β-catenin translocates to the nucleus. The ultimate interaction and binding between the nuclear β-catenin and T cell factor/lymphoid enhancer factor (TCF/LEF) is what results in the wide variety of changes in gene expressions responsible for stem cell proliferation and renewal [107,108,109] (Figure 2).

The initial discovery of Wnt canonical signaling was associated with its role in tumorigenesis when the transcriptional activation of molecule Int 1 was found to induce mammary hyperplasia in mice through pro-viral insertion into the Wnt 1 locus [110,111]. Further studies revealed that the dysregulation of the Wnt/β-catenin pathway was a catalyst for the events in the pathogenesis of colorectal carcinoma. Mutations in the APC gene result in the loss of reorientation of the Wnt destruction complex, thereby initiating carcinogenesis [112]. The role of the Wnt/β-catenin pathway in ovarian cancer has been delineated due to its persistence in the XX gonad during gonadal development and its ability to regulate stemness in the ovarian stem cell niche [105,113]. The process of epithelial to mesenchymal transition (EMT) is one that has been rigorously researched for decades in the context of tumor progression. Epithelial to mesenchymal transition is known to generate hybrid E/M cells exhibiting cell plasticity, a property that has also been observed in normal ovarian surface epithelium [114,115]. These intermediate EMT states were subsequently found in ovarian cancer cell lines and in malignant ascites, suggesting the metastatic dissemination of cancer cells [105,116]. Through the reversible transition of cancer stem cells from epithelial phenotype to a more motile mesenchymal state, they acquire features such as stemness, invasion and resistance to therapy [116]. Slug and Snail are EMT regulating transcription factors. Ovarian cancer cell lines with an increased Snail/E-cadherin ratio were found to have more aggressive therapy-resistant characteristics [105,117]. A study by Zhao-Qui Wu [118] demonstrated the role of the Wnt/canonical axis in regulating Snail and Slug expressions, in turn suppressing BRCA1 expression. These processes urged researchers to shift focus to targeting the Wnt/β-catenin pathway and its antagonists/inhibitors as a potential treatment in advanced and chemo-resistant ovarian cancers.

## 8. Wnt Inhibition by sFRP4 Micropeptides: Potential Breakthrough in Ovarian Cancer Treatment?

The regulation of ovarian tumorigenesis by Wnt normally occurs extracellularly through its antagonists [119,120]. In cancers, the dysregulated Wnt pathway affects the negative feedback mechanism resulting in defective inhibitory regulation. Some of the known antagonists are sFRP, WIF-1 and Dickkopf proteins, among others [121]. They offer a mechanism by which hyper-activated Wnt signaling is suppressed.

sFRP4 is a glycoprotein modulator and the largest member of the sFRP family. It contains two domains: a cysteine-rich domain (CRD) that is homologous to the Wnt binding site of frizzled proteins and a netrin-like domain (NLD). It has apoptotic and anti-angiogenic properties, thus acting as a tumor suppressor [122]. sFRP4 was identified as a potent anti-CSC agent in several cancers such as breast, prostate, gliomas and ovarian cancer cell lines [123]. Pohl et al. demonstrated selective apoptotic response in tumor endothelium when SFRP was administered therapeutically [122]. In a study conducted in 2012 by Saran et al., sFRP4 levels were increased in chemosensitive ovarian cancer cell line A2780, and overexpression with sFRP4 resulted in sensitization to cisplatin in chemoresistant ovarian cancer cell line A2780Cis [120]. In recent years, various studies have clearly established the role of sFRP4 in targeting ovarian CSCs. sFRP4 has been shown to chemo-sensitize ovarian CSCs for the drug cisplatin, along with activating apoptosis via increased caspase 3/7 activity [123]. Similar studies have shown that the activation of sFRP4 by the inhibition of miR-181a has resulted in reducing cisplatin resistance and stemness in high-grade serous ovarian tumors (HGSOC) [124].

**Figure 3:**
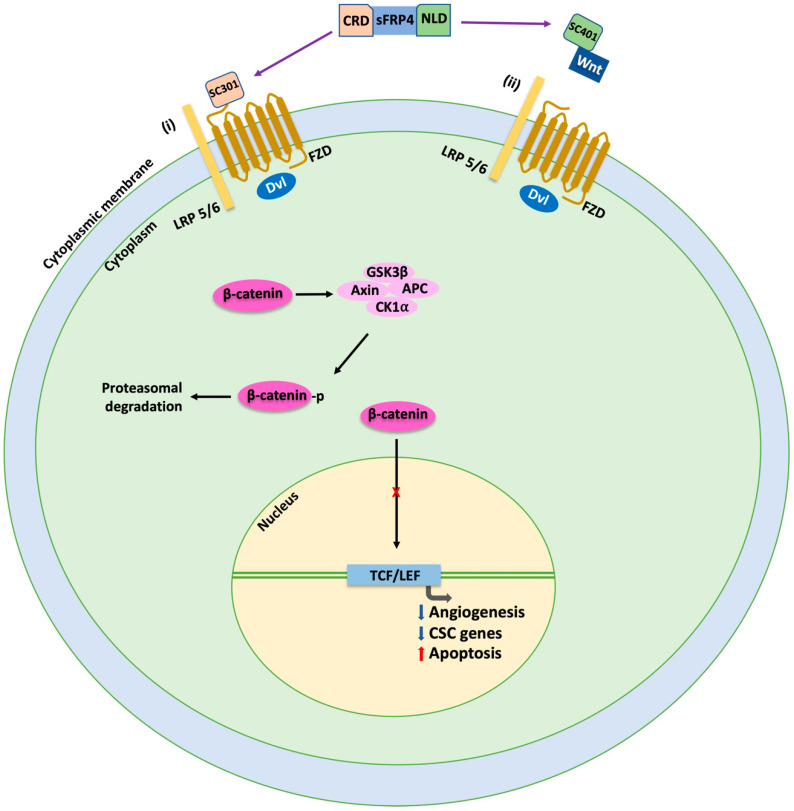
Differential inhibition of Wnt β-catenin-dependent signaling pathway by sFRP4 and its micropeptides. sFRP4 antagonizes Wnt β-catenin-dependent signaling pathway by (i) binding to FZD via its cysteine-rich domain (CRD) and (ii) binding with Wnt via its netrin-like domain (NLD). SC301 derived from CRD domain binds to FZD, whereas SC401 derived from NLD domain binds with Wnt. Inhibition of Wnt signaling leads to phosphorylation of β-catenin and its subsequent proteasomal degradation. Lack of β-catenin–TCF/LEF interaction in the nucleus causes suppression of angiogenesis and cancer stem cell (CSC) genes along with activation of apoptosis. CRD, Cysteine-rich domain; CSC, Cancer stem cell; NLD, Netrin-like domain.

In our recently published report [125], it was observed that CSCs enriched from ovarian cancer cell lines PA-1 and SKOV-3 show a high expression of CSC markers such as CD24, CD44, Nanog, Oct4, ABCG2, ABCC2 and ABCC4. In this study, we used two synthetic micropeptides, the SC-301 of 17 amino acids and SC-401 of 20 amino acids, derived from the CRD and NLD domains, respectively, of sFRP4. Treatment with these micropeptides reduced the CSC marker expression and sensitized ovarian CSCs to cisplatin. Furthermore, there was upregulation of caspases, p53 and other key pro-apoptotic genes in addition to the expected inhibition of the Wnt/β-catenin pathway. In addition, it also significantly retarded the cell migration capacity of CSCs. Our study demonstrated novel protein interaction between β-catenin and CD24 in ovarian cancer, which was disrupted upon treatment with sFRP4 micropeptides. As CD24 is known to promote cell invasion and migration, this disruption could possibly attenuate these properties. We also performed an in vivo angiogenic chorioallantoic membrane (CAM) assay and it was shown that the treatment of sFRP4-derived micropeptides inhibited the angiogenic potential of ovarian CSCs, thereby indicating that these micropeptides are highly anti-angiogenic in vivo, a valuable property for tumor suppression. The micropeptides were also potent in suppressing autophagy, which is required for CSC survival [126]. The overall findings clearly demonstrate that sFRP4 micropeptides not only inhibit the Wnt/β-catenin pathway but also may play a key role in targeting chemoresistance, cell invasion and autophagy in ovarian CSCs (Figure 3). 

## 9. Conclusions

To summarize, in this review we describe the existing prognostic and diagnostic markers in ovarian cancers and their level of specificity. The treatment strategies currently adopted by physicians globally shed light on the need for a more effective and precise panel of drugs, since relapses still remain alarmingly high. The significance of cancer stem cells in promoting chemoresistance and metastasis and the specific markers of CSCs and CTCs are described. The role of Wnt in regulating CSCs is further elaborated as the Wnt signaling pathway is one of the major determinants of the survival of CSCs. Novel targeted therapy addressing upregulated Wnt signaling would be the way forward to inhibit ovarian cancer stem cells. A natural antagonist of Wnt, the secreted frizzled related protein 4 has been well reported to increase chemotherapeutic response and decrease the CSC population. We describe how the short active peptide derivatives of sFRP4 could be the latest breakthrough in peptide-based drug compounds for the treatment of highly malignant and aggressive ovarian cancer.

## Figures and Tables

**Table 1:** Major subtypes of epithelial ovarian cancer and mutations associated with them.

Category	High-Grade Serous	Low-Grade Serous	Mucinous	Endometrioid	Clear Cell
**% in population**	70–74	3–5	2–6	7–24	10–26
**Origin**	Fallopian tube epithelium	Fallopian tube epithelium	Unknown	Endometriosis	Endometriosis
**Associated mutations**	TP53, PIK3CA, BRCA1/2	KRAS, BRAF, ERB2	KRAS, HER 2 amplification	CTNNB1, PTEN	ARID1A, PIKC3A, CTNNB1, MSI

**Table 2:** Existing markers of ovarian cancer stem cell and their role in disease progression.

Ovarian CSC Markers	Protein Type	Role in Ovarian CSCs	Reference
CD24	Mucin type glycoprotein	Poor prognosis, EMT, self-renewal, quiescence, resistance, sphere-forming capacity	[52,53,54]
CD44	Cell-surface glycoprotein	Poor prognosis, migration, invasion, drug resistance, poor differentiation, high rate of recurrence, predictive marker for distant metastasis	[55,56,57]
CD117	Receptor tyrosine kinase	Poor prognosis	[58]
CD133	Pentaspan transmembrane glycoprotein	Sphere-forming capacity, increased tumorigenic capacity	[54,59]
CD9	Tetraspanins	Poor prognosis, induces cell growth, activates NF-κB-signaling pathway	[60,61]
CD105	Endoglin (ENG)- Type I membrane glycoprotein	Drug resistance, advanced disease stage, poor differentiation, high rate of recurrence, metastasis	[56,62]
CD106	Vascular cell adhesion molecule	Drug resistance, advanced disease stage, poor differentiation, high rate of recurrence	[56]
ALDH1	Cytosolic isoform of acetaldehyde dehydrogenase	Chemoresistance, invasion, colony formation	[63,64]
OCT4	Transcription factor	Drug resistance, proliferation, activates JAK/STAT signaling pathway, angiogenesis, metastasis	[65,66]
SOX2	Transcription factor	Spheroid formation, cell proliferation, cell migration, chemoresistance, tumorigenicity, stemness, relapse	[67,68]
NANOG	Transcription factor	Poor prognosis, migration, invasion	[69,70]
ROR1	Receptor tyrosine kinases	Self-renewal, chemoresistance	[64,71]
ABCG2	ATP-binding cassette transporter	Drug resistance, self-renewal, proliferation	[72]
ABCC1	ATP-binding cassette transporter	Grading of cancer	[73]
ABCC4	ATP-binding cassette transporter	Relapse, chemoresistance	[73,74]
NESTIN	Type VI intermediate filament protein	Chemoresistance, poor prognosis, angiogenesis	[75,76]
SCF	Ubiquitin ligases	Promote stemness properties	[77]
NOTCH1	Type 1 transmembrane protein	Prognosis, Sphere formation, drug resistance, modulates expression of genes such as SOX2, ALDH and ABC transporters	[78,79]
Bmi-1	Member of the Polycomb repressor complex 1	Prognosis, cell growth, metastasis, anti-apoptotic function, chemoresistance	[80,81]
CXCR4	G-coupled chemokine receptor	Maintaining stemness, prognosis	[82,83]
EpCAM	Epithelial cell adhesion/activating molecule	Chemoresistance, metastasis, maintenance of stemness, tumor initiation	[84,85,86]
SSEA4	Sialyl-glycolipid	Advanced tumor stage, poorer tumor cell differentiation	[87]
EPHA1	Receptor tyrosine kinase	Tumor aggressiveness, proliferation, invasion, migration	[60,88]
